# *APOE* targeting strategy in Alzheimer’s disease: lessons learned from protective variants

**DOI:** 10.1186/s13024-022-00556-6

**Published:** 2022-08-03

**Authors:** Guojun Bu

**Affiliations:** Molecular Neurodegeneration, Jacksonville, USA

The *APOE* gene is the strongest genetic risk factor for Alzheimer’s disease (AD) with the ε4 allele increasing the risk by 2-4 folds compared to the most common ε3 allele [1]. The high prevalence of *APOE4* allele frequency (~ 14% in Caucasian non-demented versus ~ 38% in AD) and carrier frequency (~ 20-30% in Caucasian non-demented versus 50-70% in AD) in AD patients presents a strong rationale to target *APOE* for AD prevention and therapy [1]. More interestingly, carriers of the *APOE2* allele have a significantly reduced risk for AD [2], further attracting interests to target this strong AD risk gene.

The apolipoprotein E (apoE) protein encoded by the *APOE* gene is a major lipid carrier in both periphery and the brain transporting and distributing lipids across cells and tissues with recognized roles in homeostasis and injury repair [1]. In AD, strong clinical and preclinical evidence suggests that the main pathway by which *APOE4* drives AD risk is to promote earlier and more abundant amyloid-β (Aβ) deposition, likely by inhibiting Aβ clearance and accelerating Aβ aggregation and amyloid seeding [1]. Although Aβ plaque is a defining pathological feature of AD, it is not sufficient to cause dementia. Efforts in targeting Aβ has generated mostly disappointing clinical outcomes which is not surprising considering that Aβ deposition occurs up to two decades before the clinical onset and that the amounts of Aβ plaque typically do not correlate with dementia. As such, targeting Aβ might need to be explored as a prevention rather than a treatment strategy. Because Aβ deposition triggers or accelerates additional pathogenic events including microglia-mediated immune response and tau tangle spread, effective strategy targeting Aβ will likely be primary prevention prior to plaque development.

ApoE isoforms also have differential effects on multiple other AD-related pathogenic pathways highlighted by endocytic trafficking, immune response, cerebrovascular integrity, tau-mediated neurodegeneration, and energy metabolism [1, 3], some might be Aβ-independent. Indeed, *APOE* genotype also has differential effects on age-related cognitive decline and Lewy body dementia parallel to their risk profile for AD. How apoE isoforms that differ at only two amino acids (apoE2: Cys112 and Cys158; apoE3: Cys112 and Arg158; apoE4: Arg112 and Arg158) (Fig. 1) have such profound effects on risk for AD and other age-related dementias has puzzled the field for decades. Recently, an ApoE cascade hypothesis was proposed where it emphasizes the structural and related biochemical differences including lipidation, protein levels, receptor binding, and oligomerization, among the three isoforms as drivers of downstream effects at the cellular and phenotypical levels [4]. As such, targeting the biochemical features of apoE will likely yield greater and broader effects on multiple AD-related pathways.

**Fig. 1:**
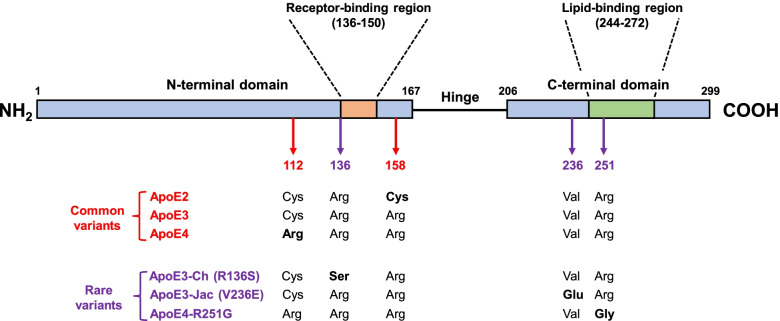
Linear structural features of apoE and the amino acid differences among common and rare variants. The numbers depict amino acid residues of the protein sequence. ApoE has three structural domains: N-terminal domain (1-167), hinge domain (168-205), and C-terminal domain (206-299). The receptor-binding region in the N-terminal domain and the lipid-binding region in the C-terminal domain are marked. ApoE3-Christchurch is abbreviated as ApoE3-Ch whereas ApoE3-Jacksonville is abbreviated as ApoE3-Jac

Although targeting strategies developed against apoE4 or learned from apoE4 are emerging including down-regulation of apoE4 levels [5], the apoE field still struggles with nominating alternative, perhaps more effective ways of targeting apoE in general. Towards this, lessons learned from studying apoE2 has provided significant insights [2]. Despite limited attention to apoE2 compared to apoE4 in preclinical studies, emerging evidence suggests that a major pathway by which apoE2 protects against AD and promotes healthy brain aging is by enhancing lipid efflux and related lipid metabolism. ApoE2 is associated with greater lipidation in CSF and higher apoE protein levels in both periphery and the brain. Interestingly, the higher apoE levels and lower cholesterol in brain parenchyma are associated with better memory performance [6] and longevity [7] in human apoE-targeted replacement mice. ApoE2 is also associated with unique lipidomic and metabolomic profiles in plasma [8, 9]. This knowledge of apoE2 highlights the potential beneficial effects of enhancing lipid efflux and promoting lipid metabolism as a lead strategy to treat AD and related dementia.

In addition to the common variants, recent studies have identified several *APOE* rare protective variants that offer new insights on apoE properties linked to their protective effects (Fig. 1). Top of the list is the *APOE3*-Christchurch (R136S) variant where a homozygous carrier with a *PSEN1* mutation had a delayed onset of mild cognitive impairment by three decades [10]. Brain imaging studies detected high amyloid load but limited tau pathology and neurodegeneration, suggesting a protective mechanism that is resilient against amyloid. One known effect of the R136S mutation is the reduced binding to the low-density lipoprotein (LDL) receptor and heparan sulfate proteoglycan (HSPG). Interestingly, several pathogenic molecules in AD including Aβ, tau, α-synuclein, and apoE bind to cell surface HSPG which has been implicated in proteinopathy in neurodegenerative diseases by modulating protein trafficking, aggregation, and propagation. How does reduced apoE binding to HSPG lightens the risk to AD is still unclear but given the detrimental effects of HSPG in pathogenic events, it is possible that targeting apoE-HSPG interaction can be beneficial to reducing dementia-related outcomes. One caution for lessons learned with the *APOE3*-Christchurch variant is that all information to date on *APOE3*-Christchurch is from study of one individual with incomplete information on neuropathological, biomarker, and other outcomes. Preclinical studies using animal models and cellular models are needed to address specific effects of this variant on neuropathological features (i.e., amyloid plaques and tau tangles), immune response, lipid metabolism, vascular integrity and function, and other AD-related pathways. It is interesting to note that both *APOE3*-Christchurch and *APOE2* homozygosity are associated with Type III hyperlipoproteinemia. Additionally, apoE2 is deficient in binding to the LDL receptor and has the lowest affinity to heparin compared to apoE3 and apoE4. As such, it is templating to speculate that the protective mechanisms of *APOE3*-Christchurch and *APOE2* are likely related in pathways relevant to receptor binding and lipid metabolism.

Another *APOE* protective variant is *APOE3*-Jacksonville (V236E) with the mutation localizing in the apoE C-terminal domain, which is best known for apoE lipid-binding function. The protective effect was first reported in 2014 [11] but only recently the underlying mechanism was revealed as reducing apoE self-aggregation [12]. Specifically, the apoE3-Jacksonville mutation reduces the tendency to aggregate both in mammalian cells and when produced in bacteria. More importantly, reduced apoE aggregation is associated with greater ability to promote cholesterol efflux, a process thought to require the apoE monomeric form. ApoE3-Jacksonville is also associated with enhanced lipid-association and when expressed in an amyloid mouse model, reduces amyloid load and related toxicity [12]. These mechanistic insights suggest that reducing apoE self-aggregation can be an alternative apoE targeting strategy to enhance apoE protective effect against AD. Reduced apoE aggregation likely alleviates the detrimental effect of apoE in amyloid seeding and promotes endocytic trafficking of apoE-interacting receptors such as apoE receptor 2 (ApoER2/LRP8), glutamate receptors, insulin receptor, and lipid-efflux transporter ABCA1. More importantly, as intracellular lipid accumulation has been increasingly recognized as a major pathogenic event in aging and AD [13], the potentially enhanced function of apoE in lipid efflux will likely promote repair and healthy brain aging.

More recently, Le Guen et al reported genetic identification of two rare *APOE* protective variants [14]. In addition to confirming the protective effect of *APOE3*-Jacksonville, they discovered a new protective variant *APOE4*-R251G which co-segregates with the *APOE4* allele. By analyzing multiple cohorts and combining data for meta-analysis, the genetic data are in general convincing despite the typical difficulties in genetic association studies on rare variants. As with *APOE3*-Jacksonville, the *APOE4*-R251G is also localized in the C-terminal domain of apoE (Fig. 1). Specifically, the R251G mutation is located within the lipid-binding region, perhaps just outside the area where apoE initially binds lipid. How this specific mutation is protective against AD risk is not clear, but two possibilities worth exploring. First, the change from a hydrophilic residue to a non-polar residue could enhance lipid binding thus promoting apoE function in lipid metabolism as it relates to brain homeostasis and injury repair. Second, the R251G mutation could potentially change the apoE4 structure such that it either reduces its harmful effects or enhances its physiological functions [1]. Towards this, it is interesting to note that the R251 residue was proposed to form a salt bridge with the Q98 residue as part of apoE domain-domain interaction in a structural study of a mutated apoE3 [15]. Collectively, the *APOE3*-Jacksonville and *APOE4*-R251G protective variants offer new clues as to how apoE C-terminal domain impacts AD and related dementias and how knowledge learned from them can guide new apoE-targeting strategies.

Although *APOE* common variants, *APOE2* and *APOE4*, continue to teach us how apoE-related outcomes contribute to AD pathogenesis, functional rare variants such as *APOE3*-Christchurch, *APOE3*-Jacksonville, and *APOE4*-R251G can provide additional insights. The fact that these rare variants carrying mutations in different regions of apoE offers additional opportunities to explore how structural and related biochemical properties of apoE impact its pathophysiology in aging and AD. Along with the common *APOE2* variant, these *APOE* rare protective variants can teach us how to target apoE as we seek effective ways to prevent and cure AD and other age-related dementias.

## Data Availability

Not applicable.

## Abbreviations

Aβ: Amyloid-β
AD: Alzheimer’s disease
ApoE: Apolipoprotein E
Ch: Christchurch
HSPG: Heparan sulfate proteoglycan
Jac: Jacksonville (Jac)
LDL: Low-density lipoprotein
